# Safety of Porcine Reproductive and Respiratory Syndrome Modified Live Virus (MLV) vaccine strains in a young pig infection model

**DOI:** 10.1186/1297-9716-44-115

**Published:** 2013-12-05

**Authors:** Francisco Javier Martínez-Lobo, Laura Carrascosa de Lome, Francisco Díez-Fuertes, Joaquim Segalés, Carlos García-Artiga, Isabel Simarro, José María Castro, Cinta Prieto

**Affiliations:** 1 Departamento de Sanidad Animal, Facultad de Veterinaria, Universidad Complutense de Madrid, Avda. Puerta de Hierro s/n, 28040 Madrid, Spain; 2 Centre de Recerca en Sanitat Animal (CReSA), UAB-IRTA, Campus de la Universitat Autònoma de Barcelona, 08193 Bellaterra, Barcelona, Spain; 3 Departament de Sanitat i d’Anatomia Animals, Universitat Autònoma de Barcelona (UAB), 08193 Bellaterra, Barcelona, Spain; 4 Departamento de Fisiología Animal, Facultad de Veterinaria, Universidad Complutense de Madrid, Avda. Puerta de Hierro s/n, 28040 Madrid, Spain

## Abstract

The objective of this study was to compare the safety of all modified live virus vaccines commercially available in Europe against Porcine Reproductive and Respiratory Syndrome Virus (PRRSV) under the same experimental conditions. For this purpose, one hundred and twenty three-week-old piglets, divided into five groups, were used. On day 0 of the experiment, nine pigs per group were removed and the remaining fifteen were vaccinated with the commercial vaccines Ingelvac PRRS MLV, Amervac PRRS, Pyrsvac-183 and Porcilis PRRS by the IM route or were mock vaccinated and used as controls. On day 3, the nine unvaccinated pigs were re-introduced into their respective groups and served as sentinel pigs. Clinical signs were recorded daily and lung lesions were determined on days 7, 14 and 21, when 5 vaccinated pigs per group were euthanized. Blood samples and swabs were taken every three days and different organs were collected at necropsy to determine the presence of PRRSV. None of the vaccines studied caused detectable clinical signs in vaccinated pigs although lung lesions were found. Altogether, these results indicate that all vaccines can be considered clinically safe. However, some differences were found in virological parameters. Thus, neither Pyrsvac-183 nor Porcilis PRRS could be detected in porcine alveolar macrophage (PAM) cultures or in lung sections used to determine PRRSV by immunohistochemistry, indicating that these viruses might have lost their ability to replicate in PAM. This inability to replicate in PAM might be related to the lower transmission rate and the delay in the onset of viremia observed in these groups

## Introduction

Porcine Reproductive and Respiratory Syndrome (PRRS) is an economically significant disease of pigs that causes respiratory distress in piglets and reproductive failure in sows [1,2]. The causal agent, PRRS virus (PRRSV), is a small, enveloped, single-stranded positive-sense RNA virus of the *Arteriviridae* family [3]. Although, in general, PRRS is clinically similar in North America and Europe, the respective strains differ in virulence [4,5] and in antigenic [6,7] and genetic [8] properties. These differences have led to the classification of PRRSV isolates into two genotypes: type 1 that comprises viruses related to the European prototype Lelystad-virus and type 2 that includes viruses related to the American prototype strain VR-2332 [8].

The huge impact of PRRS in the swine industry has stimulated the development of various types of vaccines, including inactivated and modified-live virus (MLV) vaccines, for the control of the disease in both growing pigs and breeding females. MLV vaccines based on type 1 and type 2 viruses were originally developed for the control of PRRS in growing pigs, although some of them are now registered for the control of the reproductive form of PRRS. However, the safety of these products has been questioned based on the results of some experimental studies and on field evidence. Thus, experimental studies carried out with Ingelvac PRRS MLV, a vaccine based on a type 2 isolate, have demonstrated that vaccine virus replicates in vaccinated pigs, causes detectable viremia, persists in the organism of vaccinates for weeks [9-11] and is shed by different routes causing the infection of sentinel pigs [12]. In addition, the virus can cross the placental barrier in pregnant sows infecting the developing fetuses [13] and can be transmitted to naïve newborn piglets during lactation [14]. Even more, reversions to virulence have been suspected in the field based on the similarity between the vaccine strain and some strains that have caused clinical problems in areas where the vaccine has been used, regardless of whether the affected animals had been vaccinated or not [15,16].

Despite the knowledge in relation to the safety of type 2 Ingelvac PRRS MLV vaccine, not much information has been published about the safety of MLV vaccines based on type 1 PRRS viruses, even though they are frequently used for the control of the disease in several European countries. In fact, there are only a few reports that demonstrate that these vaccines replicate in the host causing viremia during variable periods of time both in growing pigs [17] and in breeding females [18], which can lead to transplacental infection of fetuses [18]. However, no information is available regarding the ability of these vaccine strains of being shed and transmitted to in-contact pigs.

Even more, the above mentioned studies have been carried out under different conditions, using different experimental models, i.e. growing pigs versus breeding females, and different experimental designs, with differences affecting not only the age of vaccinated pigs but also the quality and quantity of the parameters measured. Under these circumstances, the information available about the safety of different vaccines is only partial and direct comparison of the results between different experiments is not feasible, making it impossible to determine whether different vaccine strains differ in their safety properties. Consequently, the objective of the present study was to elucidate and compare the safety of all MLV vaccines commercially available in Europe under the same experimental conditions, measuring their ability to induce clinical signs and lung lesions, their replication capacity in the host, their ability to be shed by different routes and their transmissibility to naïve pigs.

## Material and methods

### Animals and facilities

One hundred and one hundred and twenty three-week-old crossbred conventional piglets seronegative for PRRSV, *Mycoplasma hyopneumoniae* and negative by PCR for porcine circovirus type 2 (PCV2) were used in this study. The animals were randomly divided into five groups (A to E) of twenty-four animals each and housed in isolation in pens with a concrete floor and an automatic watering system.

All experimental procedures were approved by the Animal Ethics Committee of the Universidad Complutense de Madrid and were conducted in accordance with the guidelines of the Good Experimental Practices (GEP) standard adopted by the European Union, which ensure the protection and welfare of the animals used in research.

### Vaccines and cell cultures

The experiments were performed with all MLV vaccines registered in Europe: Ingelvac PRRS MLV (Boehringer Ingelheim, USA); Porcilis PRRS (Merck, Sharp and Dohme Animal Health, USA, formerly Intervet S.A., The Netherlands); Amervac PRRS, (Laboratorios Hipra S.A., Spain) and Pyrsvac-183 (Laboratorios Syva, S.A., Spain). The first one belongs to type 2 PRRSV while the other three are based on subtype I of type 1 PRRSV strains, although of two different clades. Thus, Porcilis PRRS strain has been classified in clade A of subtype I while Amervac PRRS strain and Pyrsvac-183 strain belong to clade D of the same subtype [19]. The parent strains of these vaccines, i.e. VR2332 (Boehringer Ingelheim), VP-046 bis (Amervac PRRS), All-183 (Pyrsvac-183) and AD (Porcilis PRRS), were isolated in the USA in 1992, in Spain in 1992 and 1991 and in The Netherlands in 1992, respectively.

The presence of PRRSV in clinical samples obtained from experimental pigs was determined by culturing the samples in the MARC-145 cell line, a cell clone highly permissive for PRRSV derived from the MA-104 cell line [20], and in porcine alveolar macrophages (PAM), prepared and maintained as previously described [21].

### Experimental design and sample collection

On day 0 of the experiment, after an acclimatization period of 7 days, nine animals per group were removed and the remaining fifteen pigs of groups A to D were vaccinated with the commercial vaccines Ingelvac PRRS MLV, Amervac PRRS, Pyrsvac-183 and Porcilis PRRS, respectively, by the IM route following the manufacturers’ instructions. Finally, fifteen pigs of Group E were mock vaccinated using a MARC-145 cell culture supernatant as the inoculum. On day 3 of the experiment, the nine unvaccinated pigs were re-introduced into their respective experimental groups and served as sentinel pigs.

During the whole experimental period, vaccinated and in-contact pigs were examined daily for clinical signs following a previously published clinical score system [5] and rectal temperatures and food intake were recorded starting 3 days before vaccination. Blood samples were collected in serum-clot vacuum tubes on days 0, 3, 6, 9, 12, 15 and 18 of the experiment from vaccinated pigs and on days 3, 6, 9, 12, 15, 18 and 24 from in-contact pigs. Serum was obtained and stored at −80 °C until use for virus isolation and to measure antibodies against PRRSV. In addition, blood samples obtained from sentinel pigs, were used to determine the presence of PRRSV by RT-PCR [22]. Viremic sentinel pigs were removed from the principal group as soon as they were detected to be viremic. Nasal, oral and rectal swabs were obtained from vaccinated pigs on the days of blood collection, immersed in 2 mL of Dulbecco’s modified eagle’s medium (DMEM) and stored at −80 °C until processed for virus isolation. Pigs were weighed on each bleeding day. Daily weight gains (DWG) were estimated for each pig for three different periods: from day 0 to day 6; from day 7 to day 15 and from day 16 to day 21 post-vaccination (pv).

On days 7, 14 and 21 of the experiment, five vaccinated pigs per group and five controls were euthanized by an overdose of sodium pentobarbital (Dolethal, Vetoquinol, France) dosed by intravenous route (IV) and a complete necropsy was performed. Samples of lung, tonsil, thymus, ileum and submandibular, superficial inguinal, sternal, mediastinal and mesenteric lymph nodes were collected and stored at −80 °C until use for virus isolation. In addition, lung samples were taken for histopathological studies. Blood samples, nasal, oral and rectal swabs were obtained at necropsy, stored at −80 °C and processed for virus isolation. On day 24 of the experiment all in-contact pigs were euthanized.

### Virus isolation and titration

Clinical samples were processed as described elsewhere [21] and were used to inoculate monolayers of PAM and MARC-145 cells in quadruplicate. Cell cultures were incubated for 90 min at 37 °C to facilitate adsorption, washed twice with culture media and fresh DMEM supplemented with 10% fetal bovine serum (FBS) was added. Then, cell cultures were incubated for 6 days at 37 °C in a humidified atmosphere containing 5% CO_2_ and the presence of cytopathic effect (CPE) typical of PRRSV was evaluated on days 4 to 6. As a positive control, strain 5710 was added to PAM cultures to a final concentration of 10^3^, 10^2^ and 10 TCID_50_/well [21]. Only batches of PAM with a minimum sensitivity to infection of at least 50% of the wells to which 10 TCID_50_ were added were used. Virus-free DMEM or FBS were used as negative controls. If CPE was observed, RT-PCR was carried out as previously described [22] to confirm the presence of PRRSV.

Virus titers were determined as described by Prieto et al. [21]. Titers were calculated as described by Reed and Muench [23], and expressed as TCID_50_/g for tissue samples or TCID_50_/mL for serum or fluid samples.

### Serological examination

Serum samples obtained from vaccinated and in-contact pigs were examined for PRRSV-specific antibodies using a commercial ELISA test (HerdChek PRRS ELISA 2XR, IDEXX Laboratories, Inc. Westbrook, MA, USA).

### Pathological examination

At necropsy, all organs were examined with emphasis on the respiratory tract. Lungs were given a score to estimate the percentage of lung surface affected, using a scoring system previously reported to evaluate gross lung lesions [4].

Lung samples (including apical, cardiac and diaphragmatic lobes) were collected and fixed by immersion in 10% neutral buffered formalin. Subsequently, pulmonary tissues were dehydrated through graded alcohol and embedded in paraffin wax. Two consecutive 4 μm thick sections were cut. One of them was stained with hematoxylin-eosin (HE) and the other one was processed to detect PRRSV antigen by immunohistochemistry. Lung sections were examined in a blinded fashion and an estimate score of the severity of the interstitial pneumonia based on a previously published scoring system [4] was given.

### Immunohistochemistry

PRRSV immunohistochemistry was based on a previously published procedure [24], using as primary antibody monoclonal antibody 1 AC7 (Ingenasa, Madrid, Spain), specific for nucleocapsid protein (N) of both PRRSV genotypes. Antigen amount was given a ranked score of 0 to 3 as an estimate of the number of foci of positive cells per section of tissue taken from each animal (three lung pieces examined): 0 = no PRRSV-antigen-positive cells; 1 = 1–5 foci of positive cells; 2 = 5–10 foci of positive cells; 3 = >10 foci of positive cells. A focus of positive cells was defined as an area studied at 400× field magnification in which at least 3–5 positive cells were observed. The scoring was also done in a blinded fashion by the same pathologist.

### Statistical analysis

The occurrence of clinical signs over the entire post-vaccination period, gross and microscopic lung lesions at necropsy and the viral determination by immunohistochemistry were evaluated for significance using the Kruskal-Wallis’s non-parametric test and Mann–Whitney *U* test. Differences in viral load and serological (S/P ratio) values at each sampling point were analyzed using a one-way analysis of variance and the Duncan’s multiple range test. A Student’s *t* test was used to assess significance in differences in rectal temperatures prior to and after vaccination in each experimental group. Differences in the proportions of positive clinical samples and of in-contact pigs infected in each experimental group were assessed for significance by the 2-tailed Fisher’s exact test. In the evaluation of virological parameters only data of groups A to D were compared while in the evaluation of clinical or productive parameters data of all five groups were compared.

## Results

### Clinical signs and growth performance

**Figure 1:**
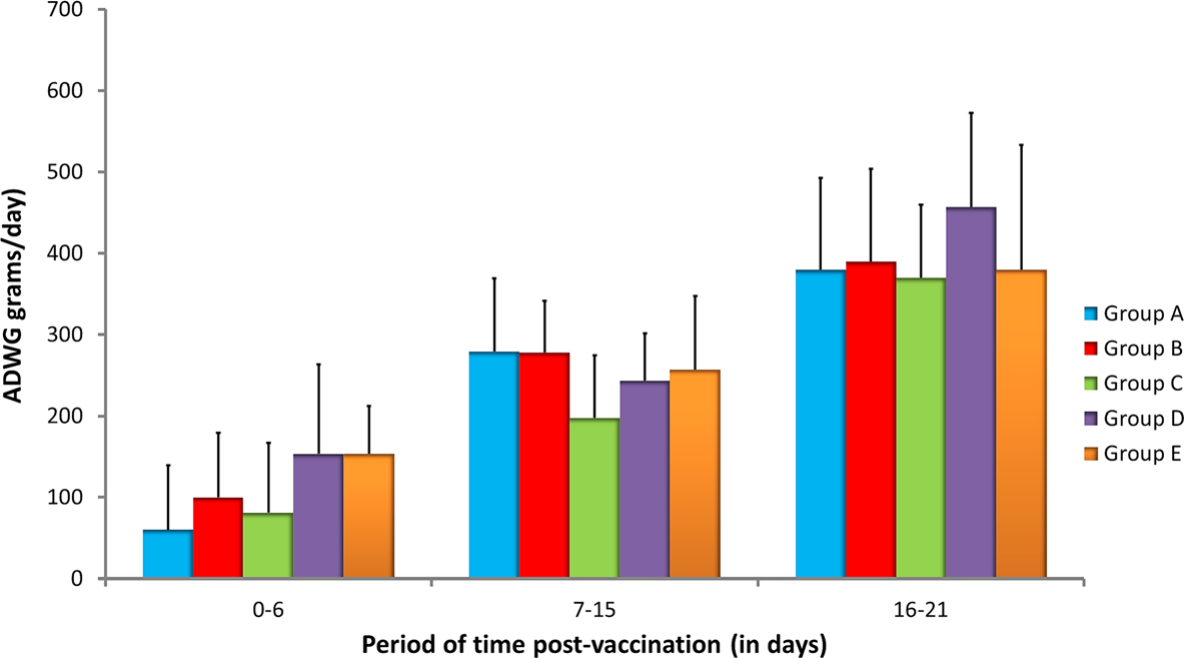
**Average Daily Weight Gain (ADWG) recorded for vaccinated pigs of all experimental groups.** All vaccinated pigs were weighted every three days. Individual weights of days 0, 6, 15 and 21 were used to estimate the ADWG of each group in periods 0 to 6; 7 to 15 and 16 to 21.

Control pigs and vaccinated animals of groups A, B, C and D remained in good health and condition after vaccination and no local or general adverse reactions were observed. Thus, no clinical signs were recorded for any pig during the experiment. Rectal temperatures remained within physiological values during the whole experimental period and no statistically significant increases were observed after vaccination in any group. All in-contact pigs remained in normal health and condition throughout the experiment, and neither clinical signs nor fever were recorded. Average DWG (ADWG) for each experimental group in the different periods analyzed is depicted in Figure 1. As observed in the figure, ADWG increased over time, regardless of the group considered. However, no statistically significant differences were observed between groups at any time.

### Macroscopic lung lesions

Gross lung lesions were never recorded in pigs of Group E, regardless of the day of necropsy. On the contrary, lesions characterized by multifocal, tan-mottled areas of pneumonia were found in all other experimental groups. However, there were significant individual variations. Thus, in some pigs no lesions were recorded and in others lesions were very mild. The mean percentages of affected lung surface in pigs of each experimental group at each necropsy day are shown in Table 1. The percentage of affected lung surface was dependent on the vaccine strain used as the inoculum. Pigs exposed to type 1 PRRSV showed mild (Groups B and C) to insignificant (Group D) lung lesions on day 7 pv with percentages of affected lung surface ranging from 0.7% to 2.4%. These lesions tended to resolve in the following days, leading to unremarkable lung lesions on day 21 pv. On the contrary, pigs of Group A, vaccinated with a type 2 PRRS vaccine strain, had the less extensive lung lesions on day 7 pv and afterwards the percentage of affected lung surface increased, reaching its maximum values on day 21 pv. However, when the percentages of affected lung surface were compared, no statistically significant differences were observed between groups.

**Table 1:** Macroscopic lung lesion scores recorded for each experimental group in each day of necropsy.

**Groups**	**Necropsy day**		
	**Day 7 pv**	**Day 14 pv**	**Day 21 pv**
A	0.5 ± 0.9^a^	1.4 ± 1.1	2.3 ± 2.4
B	2.2 ± 1.6	1.6 ± 2.2	0.6 ± 0.9
C	2.4 ± 3.3	0.0 ± 0.0	0.0 ± 0.0
D	0.7 ± 0.8	0.7 ± 1.1	0.0 ± 0.0
E	0.0 ± 0.0	0.0 ± 0.0	0.0 ± 0.0

^a^ mean ± standard deviation of macroscopic lung lesion scores.

### Microscopic lung lesions

No microscopic lung lesions were recorded in any of the control pigs. However, some pigs of all other experimental groups developed interstitial pneumonia characterized by multifocal to diffuse, slight to moderate thickening of the alveolar wall with variable amounts of macrophages between alveoli. The severity of the interstitial pneumonia observed in vaccinated pigs globally increased over time, peaking the mean score values at 21 days pv. The number of pigs with microscopic lesions and the mean pathological score plus standard deviations of all experimental groups at different necropsy days are given in Table 2. The number of pigs showing microscopic lung lesions was lower in groups C and D than in groups A and B. However, differences between groups were statistically significant only when groups A and B were compared to groups C and E on day 21 pv (*P* < 0.05). Likewise, the severity of the lesions recorded differed between groups. Thus, lesions were classified as moderate (score 2) or mild (score 1) in pigs of groups A and B while they were always mild (score 1) in pigs of groups C and D. However, differences in the severity of the microscopic lung lesions were statistically significant only when Group A was compared to groups C, D and E (*P* < 0.05) and when Group B was compared to groups C and E (*P* < 0.05) on day 21 pv.

**Table 2:** Microscopic lung lesion scores recorded for each experimental group in each day of necropsy.

**Group**	**Necropsy day**		
	**Day 7 pv**	**Day 14 pv**	**Day 21 pv**
A	0.2 ± 0.4^a^	0.0 ± 0.0	1.6 ± 0.5
B	0.0 ± 0.0	0.4 ± 0.5	1.2 ± 0.4
C	0.0 ± 0.0	0.0 ± 0.0	0.2 ±0.4
D	0.2 ± 0.4	0.0 ± 0.0	0.4 ± 0.5
E	0.0 ± 0.0	0.0 ± 0.0	0.0 ± 0.0

^a^ mean ± standard deviation of microscopic lung lesion scores.

### Detection of PRRSV in vaccinated pigs

#### Viremia

Viremia was never detected in any control pig, regardless of the cell culture type used as support for viral replication. The results of virus isolation from serum samples collected at different days post-vaccination from pigs of groups A to D are summarized in Table 3. All vaccine strains induced viremia in vaccinated pigs, although the number of positive pigs varied depending on the group, the day considered and the cell type used for virus isolation. When virus isolation was attempted on MARC-145 cell cultures most animals were positive from day 3 onwards on groups A and B, while viremia was detected only from day 6 onwards in pigs of groups C and D. The differences in the onset of viremia were statistically significant (*P* < 0.05). Even more, the total number of positive serum samples obtained after vaccination varied depending on the group considered (i.e. 96% for pigs of Group A; 88% for pigs of Group B; 64% for pigs of Group C and 57.3% for pigs of Group D). When virus isolation was carried out on PAM cultures most of the samples tested were negative, regardless of the group considered. Only three samples from pigs of Group A (4.0%) and five samples from pigs of Group B (6.67%), all of them obtained from 12 days pv onwards, were positive.

**Table 3:** Results of virus isolation from serum samples of vaccinated pigs in MARC-145 and PAM cultures.

**Days post-vaccination**	**Virus isolation on Marc-145**				**Virus isolation on PAM**			
	**Group**				**Group**			
	**A**	**B**	**C**	**D**	**A**	**B**	**C**	**D**
0	0/15 (0) ^a^	0/15 (0)	0/15 (0)	0/15 (0)	0/15 (0)	0/15 (0)	0/15 (0)	0/15 (0)
3	12/15 (80)	14/15 (93.3)	0/15 (0)	0/15 (0)	0/15 (0)	0/15 (0)	0/15 (0)	0/15 (0)
6	15/15 (100)	14/15 (93.3)	14/15 (93.3)	11/15 (73.3)	0/15 (0)	0/15 (0)	0/15 (0)	0/15 (0)
7	5/5 (100)	5/5 (100)	5/5 (100)	3/5 (60)	0/5 (0)	0/5 (0)	0/5 (0)	0/5 (0)
9	10/10 (100)	8/10 (80)	6/10 (60)	8/10 (80)	0/10 (0)	0/10 (0)	0/10 (0)	0/10 (0)
12	10/10 (100)	8/10 (80)	9/10 (90)	8/10 (80)	1/10 (10)	3/10 (30)	0/10 (0)	0/10 (0)
14	5/5 (100)	4/5 (80)	3/5 (60)	4/5 (80)	1/5 (20)	1/5 (20)	0/5 (0)	0/5 (0)
15	5/5 (100)	5/5 (100)	3/5 (60)	3/5 (60)	0/5 (0)	0/5 (0)	0/5 (0)	0/5 (0)
18	5/5 (100)	4/5 (80)	4/5 (80)	4/5 (80)	0/5 (0)	1/5 (20)	0/5 (0)	0/5 (0)
21	5/5 (100)	4/5 (80)	4/5 (80)	2/5 (40)	1/5 (20)	0/5 (0)	0/5 (0)	0/5 (0)

^a^ Number of positive samples/Number samples tested (Percentage of positive samples).

**Figure 2:**
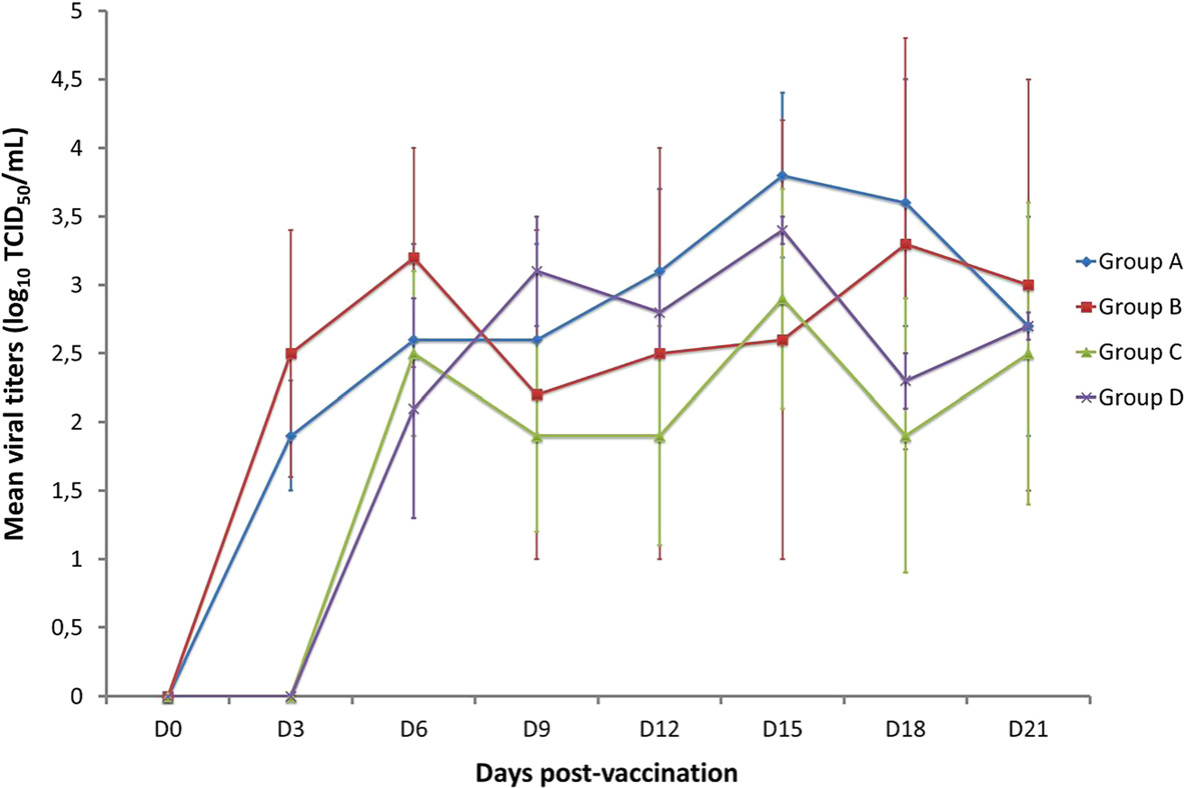
**Mean viral titers in positive serum samples of vaccinated pigs at each sampling day.** Titers are expressed as tissue culture infectious doses 50 (TCID_50_) per mL of serum. Bars represent the standard deviation.

The mean viral titers of serum samples positive by virus isolation in MARC-145 cell cultures were calculated and are depicted in Figure 2. Viral titers varied slightly depending on the group and day of the experiment considered. The highest mean viral titers in serum samples were obtained in pigs of Group A in the last week of the experiment. On the contrary, mean viral titers tended to be lower in serum samples of pigs of Group C than in any other group during the whole experimental period. However, no statistically significant differences were observed in mean viral titers between groups at any time. Viral titers were also calculated for serum samples positive in PAM cultures. The mean titer of those samples was very low, barely above the detection limit (data not shown).

#### Organic distribution

The results of virus isolation in MARC-145 cell cultures from different tissue samples collected at different days of necropsy from vaccinated pigs of Groups A, B, C and D are shown in Table 4. All tissue samples obtained from control pigs were negative by virus isolation. On the contrary, PRRSV was isolated from at least one tissue sample of all vaccinated pigs, regardless of the group they belonged to or the day of necropsy. However, the frequency of PRRSV isolation varied depending on the tissue sample considered. Thus, PRRSV was more frequently isolated from tonsils, with 49 out of 60 positive samples (81.7%), than from any other sample. On the contrary, the organ from which PRRSV was less frequently isolated was the spleen, with only 13 out of 60 positive samples (21.7%).

**Table 4:** Results of virus isolation from tissue samples of vaccinated pigs in MARC-145 cultures.

**Organ**	**Group A**				**Group B**				**Group C**				**Group D**			
	**Days post-vaccination**				**Days post-vaccination**				**Days post-vaccination**				**Days post-vaccination**			
	**7**	**14**	**21**	**Total**^**b**^	**7**	**14**	**21**	**Total**^**b**^	**7**	**14**	**21**	**Total**^**b**^	**7**	**14**	**21**	**Total**^**b**^
Lung	0/5^a^	3/5	5/5	53.3	3/5	4/5	3/5	66.7	0/5	1/5	2/5	20	1/5	2/5	2/5	33.3
Tonsil	5/5	4/5	5/5	93.3	4/5	4/5	5/5	86.7	4/5	4/5	5/5	86.7	2/5	4/5	3/5	60.0
Ileum	3/5	1/5	2/5	40	4/5	2/5	3/5	60.0	2/5	1/5	2/5	33.3	2/5	2/5	1/5	33.3
Thymus	1/5	0/5	3/5	26.7	2/5	3/5	3/5	53.3	1/5	0/5	2/5	20	2/5	2/5	1/5	33.3
Spleen	0/5	0/5	1/5	6.7	2/5	3/5	4/5	60.0	0/5	1/5	0/5	6.7	0/5	2/5	0/5	13.3
Mes. L.N.	3/5	0/5	0/5	20	2/5	3/5	4/5	60.0	2/5	2/5	2/5	40	1/5	1/5	0/5	13.3
Med. L.N.	4/5	1/5	2/5	46.7	5/5	2/5	4/5	73.3	4/5	3/5	3/5	66.7	2/5	3/5	0/5	33.3
L.S.L.N.	2/5	1/5	3/5	40	3/5	4/5	5/5	80	2/5	4/5	3/5	60	2/5	2/5	2/5	40.0
R.S.L.N	2/5	1/5	3/5	40	5/5	4/5	5/5	93.3	1/5	4/5	4/5	60	2/5	3/5	2/5	46.7
L.S.I.L.N.	2/5	1/5	2/5	33.3	1/5	2/5	4/5	46.7	0/5	1/5	1/5	13.3	1/5	3/5	1/5	33.3
R.S.I.L.N.	3/5	2/5	3/5	53.3	1/5	3/5	3/5	46.7	0/5	2/5	3/5	33.3	1/5	3/5	1/5	33.3
Est.L.N.	1/5	2/5	4/5	46.7	1/5	2/5	4/5	46.7	0/5	1/5	1/5	13.3	2/5	3/5	0/5	33.3
Total^b^	43.3	26.7	55.0	41.7	55.0	60.0	64.4	64.4	26.7	40.0	46.7	37.8	30.0	50.0	21.7	33.9

^a^ Number of positive samples/total number of samples; ^b^ Percentage; Mes.L.N: Mesenteric Lymph Node; Med.L.M.: Mediastinic Lymph Node; L.S.L.N.: Left Submandibular Lymph Node; R.S.LN: Right Submandibular Lymph Node; L.S.I.L.N.: Left Superficial Inguinal Lymph Node; R.S.I.L.N.: Right Superficial Inguinal Lymph Node; Est.L.N.: Esternal Lymph Node.

When the percentage of positive samples was compared between groups it was observed that the frequency of isolation of PRRSV from tissue samples was higher in samples of pigs of Group B than in any other group, being the global percentage of positive organs 64.4% compared to 41.7%, 37.8% and 33.9% in groups A, C and D, respectively. These differences were statistically significant (*P* < 0.05). However, when the frequency of isolation from different organs analyzed in the study was compared between groups, differences were not statistically significant, probably due to the low number of pigs per group. The only exception was the frequency of PRRSV isolation from spleen samples, which was significantly higher in pigs of group B (*P* < 0.05) than in any other group.

**Figure 3:**
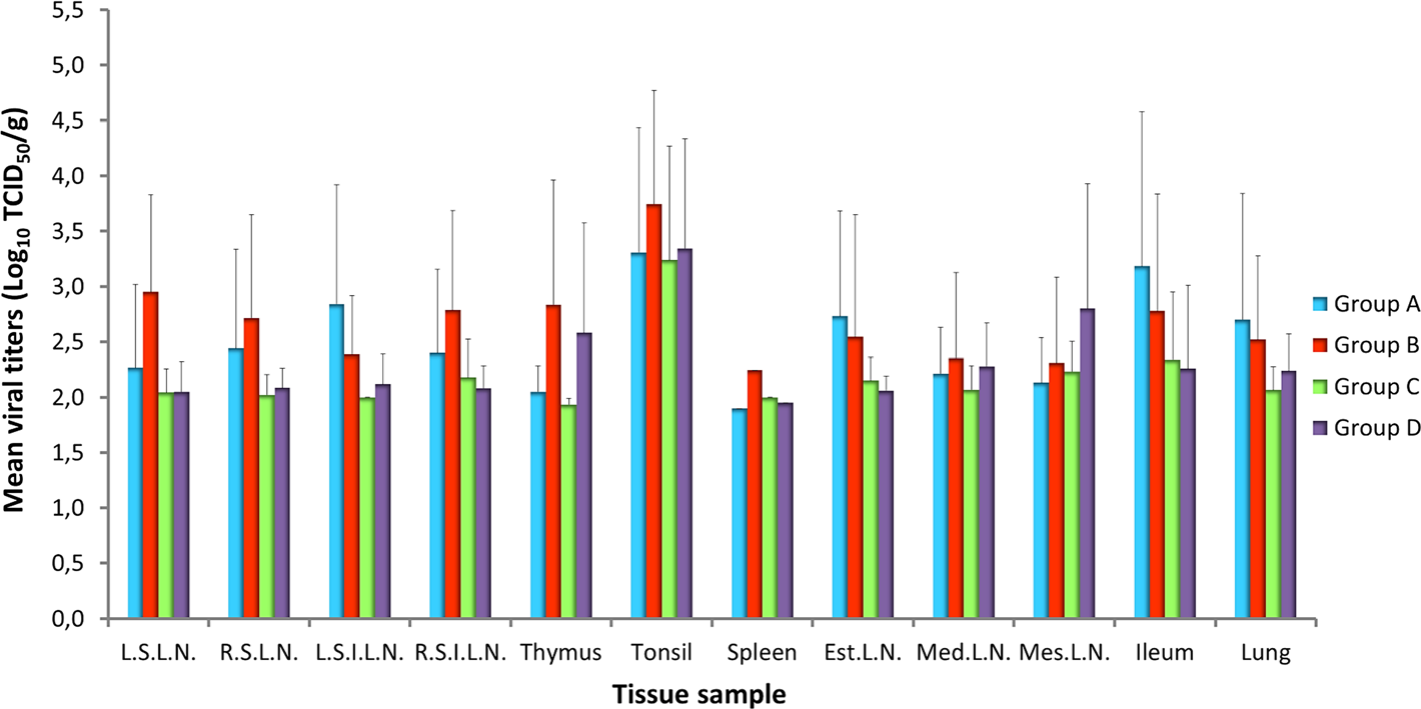
**Mean viral titers in positive tissue samples of vaccinated pigs at each necropsy day.** Titers are expressed as tissue culture infectious doses 50 (TCID_50_) per gram of tissue. Bars represent the standard deviation. LSLN: left superficial lymph node; RSLN: right superficial lymph node; LSILN: left superficial inguinal lymph node; RSILN: right superficial inguinal lymph node; Est.LN: esternal lymph node; Med.LN: mediastinic lymph node; Mes.LN: mesenteric lymph node

The viral load in tissue samples positive by virus isolation on MARC-145 was calculated and the mean values are represented in Figure 3. Viral titers tended to be higher in tonsils than in any other tissue regardless of the group considered as observed in the Figure. However, differences in viral titer between organs in each particular group and in the same organ between groups were not statistically significant.

When virus isolation was performed in PAM cultures, PRRSV was only recovered from two tonsils of animals from Group A and four tonsils of pigs from Group B, all of them on day 21 pv. The viral titer of positive samples was very low, barely above the detection limit (data not shown).

#### Immunohistochemistry

PRRSV antigen was never detected by IHC in any lung section of control pigs. However, it was detected in some samples of vaccinated pigs. Nonetheless, the number of positive pigs was very low, as was the number of positive foci found in positive samples, which led to very low mean score values. Thus, all samples from pigs of groups C and D were negative by IHC, as well as all samples obtained from pigs of groups A and B on days 7 and 14 pv. However, on day 21 pv 3 out of 5 samples obtained from pigs of Group A and 2 out of 5 samples obtained from pigs of Group B were positive by IHC, all of them with a score of 1. When PRRSV antigen was detected, it was primarily found within the cytoplasm of macrophages in the alveolar space.

#### Virus shedding

The results of PRRSV isolation from different body fluids collected at intervals after vaccination from vaccinated pigs are summarized in Table 5. As expected, all samples collected from negative controls were negative by virus isolation. However, all vaccine strains studied could be detected in all secretions analyzed, although the frequency of shedding varied slightly depending on the route considered. Thus, the presence of PRRSV was relatively common in oropharyngeal secretions from day 6 to day 15 pv and sporadic thereafter, with a total percentage of positive samples of 8.33%. On the contrary, the virus was detected more sporadically in nasal secretions, with a global percentage of positive samples of 4.67%, although shedding started earlier (i.e. day 3 pv) and lasted until day 18 pv. Finally, in feces PRRSV could be detected from day 6 pv until the end of the experiment, with a percentage of positive samples of 5.67%. Differences in the frequency and duration of shedding depended on the vaccine tested. The highest number of positive samples was obtained in pigs of Group A (18 in total, which represents 8.0% of the samples taken after vaccination) and the lowest number in Group D (11 positive samples, meaning 4.89%), being groups B and C in intermediate positions (12 and 15 positive samples, i.e. 5.33% and 6.67%, respectively). Nonetheless, differences in the number and distribution of positive samples were not statistically significant. Virus titer in positive samples was not determined.

**Table 5:** Results of virus isolation from different swabs collected from principal pigs in MARC-145 cells.

**Days of the experiment**	**Nasal swabs**				**Oral swabs**				**Rectal swabs**			
	**Group**				**Group**				**Group**			
	**A**	**B**	**C**	**D**	**A**	**B**	**C**	**D**	**A**	**B**	**C**	**D**
0	0/15^a^	0/15	0/15	0/15	0/15	0/15	0/15	0/15	0/15	0/15	0/15	0/15
3	1/15	0/15	0/15	0/15	0/15	0/15	0/15	0/15	0/15	0/15	0/15	0/15
6	1/15	0/15	1/15	2/15	2/15	1/15	3/15	1/15	0/15	0/15	0/15	0/15
7	2/5	1/5	1/5	0/5	1/5	1/5	0/5	1/5	2/5	1/5	1/5	1/5
9	0/10	0/10	0/10	0/10	1/10	0/10	2/10	1/10	2/10	2/10	1/10	1/10
12	0/10	0/10	2/10	0/10	0/10	0/10	1/10	1/10	0/10	1/10	0/10	1/10
14	0/5	0/5	1/5	0/5	0/5	3/5	1/5	0/5	0/5	0/5	1/5	1/5
15	1/5	0/5	0/5	0/5	2/5	1/5	0/5	0/5	0/5	0/5	0/5	0/5
18	0/5	1/5	0/5	0/5	0/5	0/5	0/5	0/5	1/5	0/5	0/5	0/5
21	0/5	0/5	0/5	0/5	1/5	0/5	0/5	1/5	1/5	0/5	0/5	0/5
Total	5/90	2/90	5/90	2/90	7/90	6/90	7/90	5/90	6/90	4/90	3/90	4/90

^a^ Number of positive samples/Number of samples tested.

### Seroconversion of vaccinated pigs

Pigs of Group E remained seronegative until the end of the study. Among vaccinated pigs euthanized on day 7 pv, PRRSV specific antibodies were detected only in one pig of Group C. However, several pigs of different groups were already seropositive on day 12 pv and the number of seropositive animals among those euthanized on day 14 pv was 5, 3, 4 and 5 for groups A, B, C and D, respectively. Finally, on day 21 pv all pigs, regardless of the group they belonged to, were seropositive. Despite the slight differences in the timing of seroconversion, no statistically significant differences were detected between groups in the number of seropositive pigs or in the S/P ratio values of seropositive pigs.

### Transmission of vaccine strains to sentinel pigs

#### Viremia

**Figure 4:**
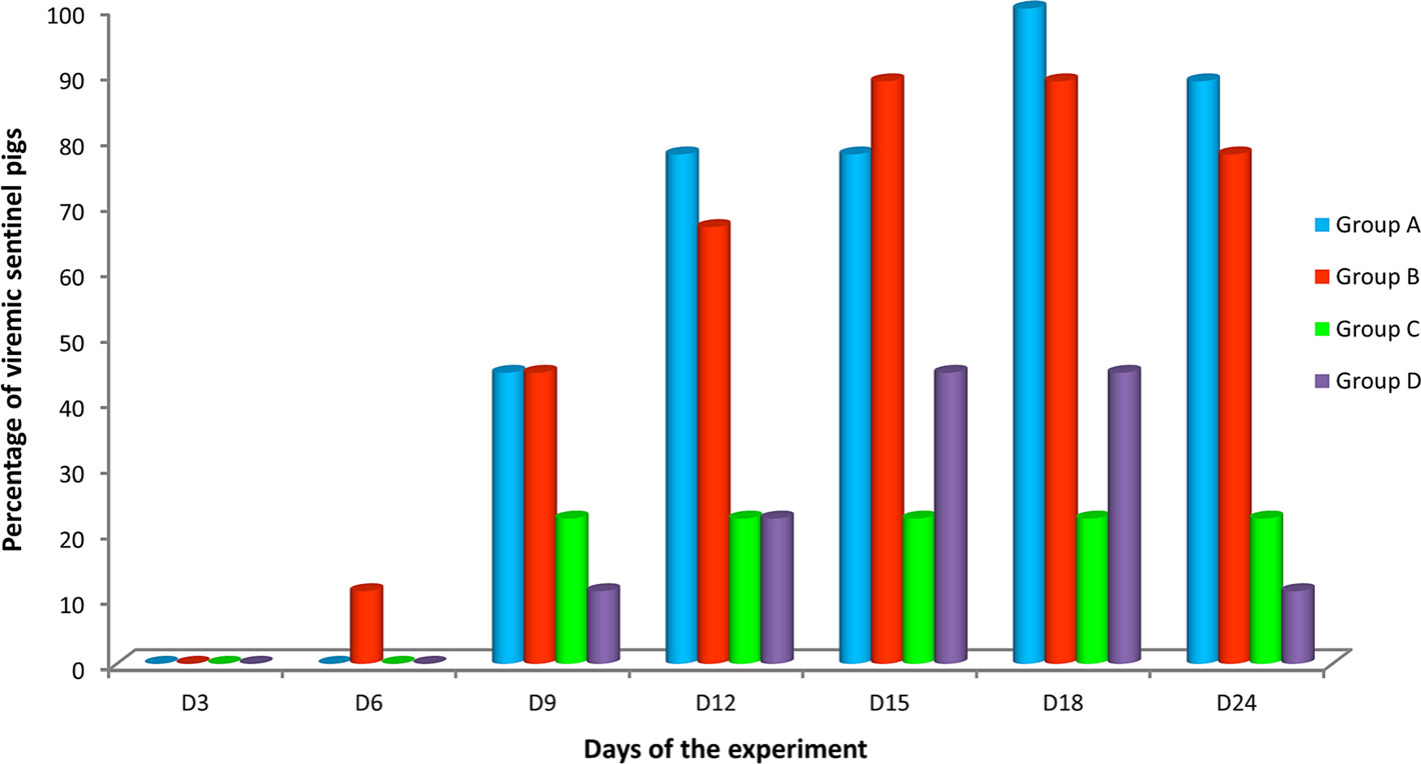
**Proportion of viremic sentinel pigs from each experimental group in each sampling day.** Blood samples were taken from sentinel pigs every three days. The percentages of sentinel pigs found viremic in each experimental group in each sampling day are represented in the figure.

The results of virus isolation in MARC-145 cell cultures from serum samples collected from sentinel pigs of groups A, B, C and D at different times of the experiment are shown in Figure 4. All vaccine strains were able to induce viremia in the sentinel pigs; however, the percentage of viremic pigs varied depending on the vaccine strain and the day of the experiment considered. Thus, viremia was detected from day 9 to the end of the experiment in at least some of the sentinel pigs of Group A and all of them were viremic on day 18. Viremia was detected earlier in pigs of Group B (one sentinel pig was already viremic on day 6 of the experiment in this group) and the virus was detected in most of the sentinel pigs from day 12 to the end of the experiment. However, there was one pig that was never viremic. On the contrary, most sentinels of groups C and D remained negative by virus isolation until the end of the experiment. In Group C only two animals (22.2%) were viremic from day 9 to the end of the experiment. In Group D viremia was detected at least once in four sentinel pigs (44.4%) and in this group viremia tended to be shorter, with only one viremic sentinel pig on day 24. The differences in the percentage of viremic sentinel pigs were statistically significant when the results of Group A were compared to the results of groups C and D on days 18 and 24 of the experiment (*P* < 0.05), and also when the results of Group B were compared to the results of Group C on day 18 (*P* < 0.05).

When virus isolation from serum samples of sentinel pigs was performed in PAM cultures, PRRSV was recovered from a limited number of samples; particularly from two pigs of Group A on day 21 of the experiment and from one pig of Group B on day 18 of the experiment.

Viral load of positive samples ranged from 1.8 TCID_50_/mL to 3.6 TCID_50_/mL, depending on the group and day of the experiment considered. However, no statistically significant differences were found between groups (data not shown).

#### Seroconversion

**Figure 5:**
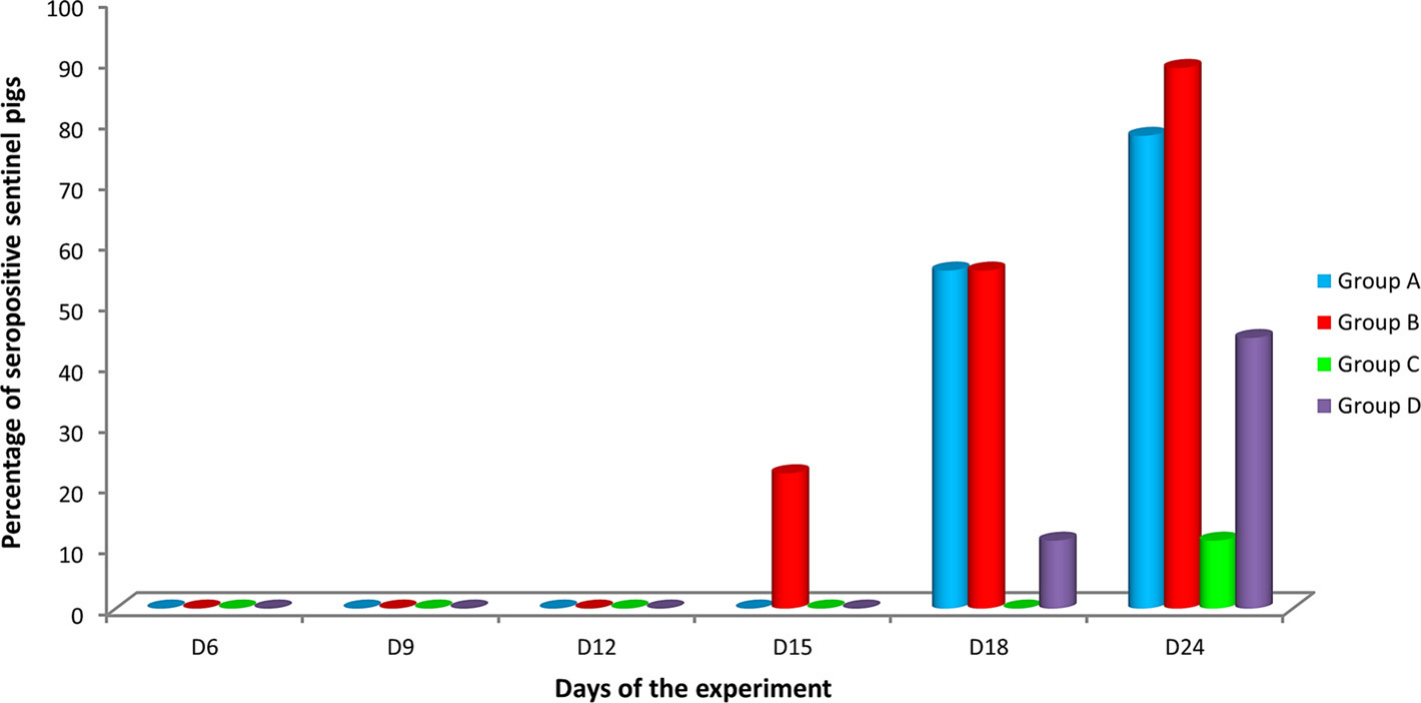
**Proportion of PRRSV seropositive sentinel pigs from each experimental group in each sampling day.** Seroconversion of sentinel pigs was assessed by ELISA test. The percentage of sentinel pigs positive by ELISA test in each experimental group in each sampling days are represented in the figure.

Seroconversion of sentinel pigs was not detected until day 15 of the experiment, when two sentinel pigs of Group B were seropositive (Figure 5). On day 18, the number of seropositive sentinel pigs in Group B had increased to five and, additionally, five sentinel pigs of Group A and one sentinel pig of Group D were seropositive. Finally, at the end of the experiment, on day 24, seven, eight, one and four sentinel pigs of groups A, B, C and D, respectively, had seroconverted.

#### Viral shedding

**Figure 6:**
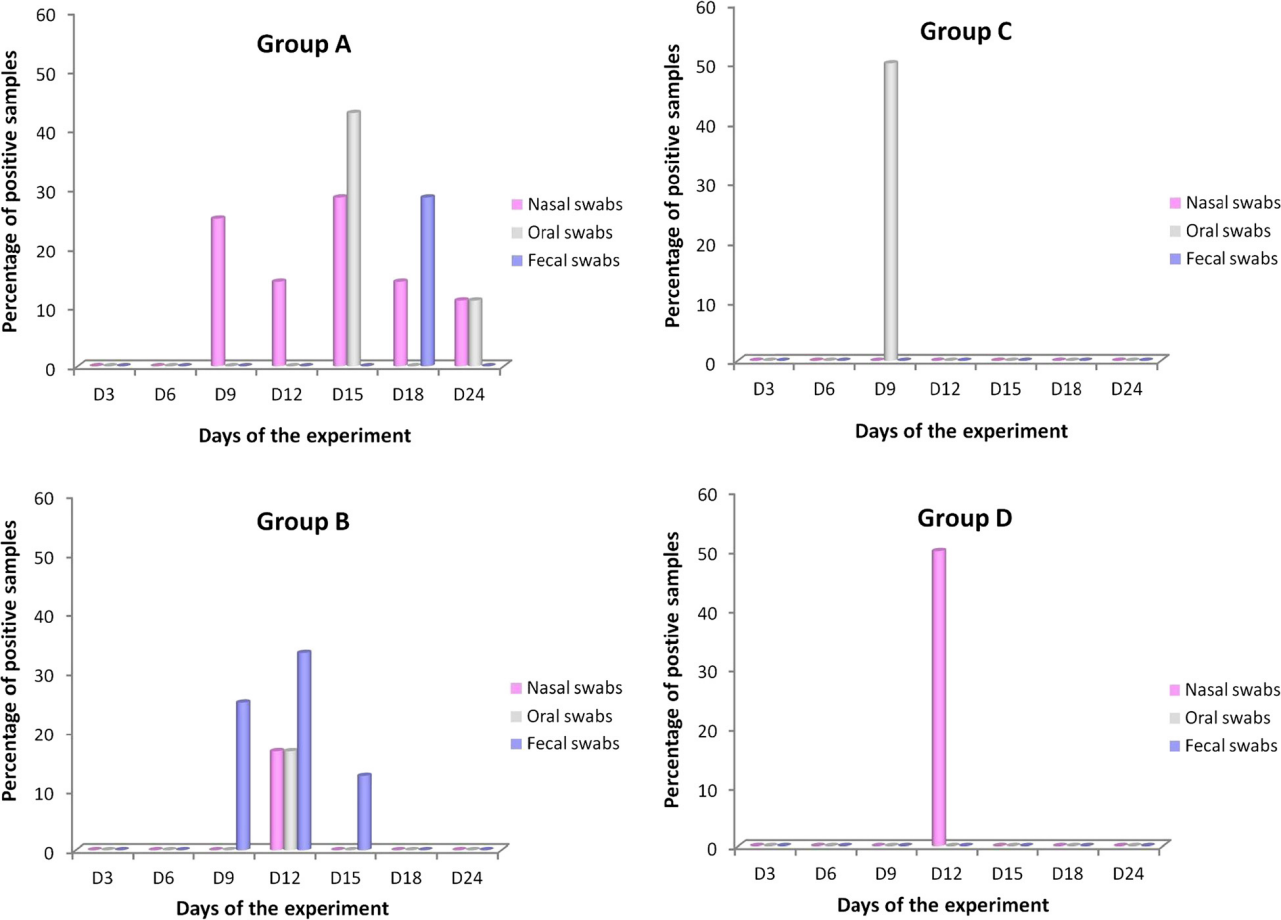
**Percentage of PRRSV positive swabs obtained from infected sentinel pigs during the experiment.** Oral, rectal and nasal swabs were obtained from sentinel pigs every three days. Swabs from sentinel pigs considered to be infected (based on the determination of viremia in the same days or in previous days) were used for virus isolation. The percentage of positive swabs obtained from infected pigs in each experimental group each sampling day is represented in the figure.

Infected sentinels shed vaccine viruses by different routes, although the frequency of shedding varied between groups and routes considered (Figure 6). Vaccine virus was detected in 12 out of 102 (11.77%) samples obtained from infected sentinel pigs of Group A, all of them between day 9 and day 24 of the experiment. In this group, the virus was more frequently shed in nasal secretions, followed by oral secretions and only sporadically in feces. On the contrary, PRRSV was more frequently isolated from fecal samples of infected sentinels of Group B than from any other sample, with 4 out of 35 positive samples, while only one nasal swab and one oral swab were positive. In total 5.71% of swabs obtained from infected sentinel pigs of Group B were positive. Finally, PRRSV was very rarely isolated from sentinel pigs of groups C and D with only one out of 30 samples (i.e. 3.33%) and one out of 45 samples (i.e. 2.22%), respectively taken from infected pigs rendering a positive result.

## Discussion

The major impact of PRRSV on pig production has stimulated the development of various types of vaccines, including inactivated virus and MLV vaccines for the control of the disease. However, only a limited number of controlled studies have been carried out to demonstrate the safety of MLV vaccines based either on type 1 or type 2 PRRSV strains [9,13,14,18,25]. Most of these studies have been designed to prove the safety of one or two commercially available vaccines and no comparative studies have ever been carried out. Moreover, the comparison of the results of the different research studies is difficult because they have used diverse experimental designs, age, breeds and health status of pigs. To obtain information that allows comparisons, we investigated the safety of four different MLV vaccine strains available in different European countries under the same experimental conditions in a young pig model (i.e. 28 day-old). Our criterion to evaluate MLV vaccine safety in this model was based on the severity of clinical signs, gross and microscopic lung lesions, viremia, viral replication in different organs and tissues, shedding of the vaccine strains and transmission rate to sentinel pigs.

The results of our study indicate that some differences can be established in the safety of the four MLV vaccines compared, although not in all parameters evaluated. Thus, no differences were observed in relation to the induction of clinical signs or in the productive performance of pigs of the different experimental groups. In fact, no clinical signs were recorded for any pig during the complete experimental period. The absence of clinical signs, and specifically of respiratory clinical signs, was consistent with the recording of slight to moderate gross lung lesions at necropsy, regardless of the group considered. Although, the percentage of affected lung surface can be considered similar in all experimental groups, in pigs exposed to type 1 MLV vaccine strains, macroscopic lung lesions resolved over time while in pigs exposed to type 2 PRRSV strain the percentage of affected lung surface increased over time until the end of the experiment. This picture is coincident with previous findings comparing type 1 and type 2 field strains that indicate that macroscopic lung lesions produced by type 2 PRRSV isolates tend to be more persistent and last longer than those produced by type 1 PRRSV isolates [5]. Thus, it is likely that the pattern of onset and resolution of lesions observed in the present study are more related to the genotype of the parent strains of the vaccine viruses than to changes occurring during the attenuation process. However, since the pathophysiologic characteristics of the parent strains of each of the vaccine strains tested in this study could not be determined, we cannot completely rule out the influence of the attenuation process in the incidence and evolution of lung lesions.

The mildness of macroscopic lung lesions recorded resulted in a low frequency and severity of microscopic lung lesions, although, on the contrary to what was observed with macroscopic lung lesions, they increased over time in all groups. The persistence of microscopic lung lesions when macroscopic changes are mild or absent observed in this study has been previously reported by others [5,24] and seems to be typical of PRRSV infection. Differences in the frequency and severity of microscopic lung lesions were statistically significant between groups on day 21 pv, when the number of pigs with lesions was the greatest and the severity of those lesions was the highest. These differences might be due to, at least, two different reasons. On the one hand, differences in the intrinsic characteristics of the parent strains and, in particular, in their pneumotropism and pathological effects in the host may be behind the differences found in the frequency and severity of microscopic lung lesions. These differences have been previously described when the virulence of different wild-type PRRSV has been compared in a young pig infection model [4,5,26]. On the other hand, the attenuation process might have changed the tropism of the viruses and limited their ability to replicate in the lung and cause lung pathology. In this line of thinking it is remarkable that the frequency of virus isolation from lung tissues found in this study was significantly lower than the frequency of virus isolation from the same organ found in similar studies carried out with wild-type viruses [5,27] or even in a study carried out in adult boars [28], indicating that the attenuation process might cause changes related to cell tropism in the lung. These changes most probably impair virus replication in PAM until a point when replication in this cell type might be completely abolished. This theory is supported by two facts. The first one is that the presence of PRRSV in PAM could only be confirmed by IHC in lung sections of some pigs of groups A and B and always on day 21 pv. The second one is that PRRSV replication was demonstrated *in vitro* in PAM cultures only in samples of some pigs of groups A and B. Even more, vaccine viruses of Pysvac-183 and Porcilis PRRS were never detected in PAM, suggesting that those vaccine viruses might have completely lost their ability to replicate in the natural target cell of PRRSV while the other two vaccine strains tested in this study can replicate, although poorly, in PAM. It could be speculated that those viruses that retain certain ability to replicate in PAM will be found in the lung more frequently and that this replication in the lung might be responsible for the development of lung lesions. Thus, the mildness of the lesions recorded might be a consequence of the limited replication of the vaccine viruses in the lung and an indicator of attenuation. However, it should be kept in mind that differences in the pneumotropism and pathogenicity of the parent viruses could also account for the differences found in the development of lung lesions in vaccinated pigs.

In order to compare viremia and viral organic distribution in pigs of different experimental groups, cultures of MAP and MARC-145 cell line were used. When the number of viremic pigs, as determined by virus isolation in MARC-145 cells, was compared between groups, the number of positive pigs in groups C and D was lower than in groups A and B, although differences were not statistically significant. However, differences between groups in the dynamics of viremia were significant. Thus, most pigs of Groups A and B were already viremic on day 3 of the experiment, while in Groups C and D the onset of viremia was delayed and the first positive animals were detected on day 6 pv. In the same way, the number of positive tissue samples was higher in pigs of groups A and B than in pigs of groups C and D. However, when the number of positive samples was compared by tissue sample and by day, in most cases, the differences were not statistically significant. This lack of significance might be partly due to the low sample size. Actually, when the total number of positive samples per group, regardless of the tissue sample or necropsy day considered, were compared, the differences were statistically significant between group B and all other groups.

When the dynamics of viremia and the organ distribution of vaccine strains were compared to those of field strains using a similar model [5], no clear differences between attenuated and wild-type viruses were found, although the organic distribution appears to be more limited in pigs exposed to vaccine strains. Thus, the percentage of positive organs in pigs exposed to wild-type viruses ranged from 65.0% to 86.7% [5] while in the present study only between 33.9% and 64.4% of the samples were positive. However, due to the existence of remarkable differences between wild-type PRRSV isolates, it is not possible to categorically state that the lower detection rate reported in this study is due to changes in the replication capacity of the vaccine viruses occurring during the attenuation process. To confirm this theory it would be necessary to determine the organ distribution of the parent strains and compare it to the attenuated viruses.

Nonetheless, and despite the lower percentage of positive organs found in this study compared to organ distribution of wild-type viruses, the organs in which PRRSV was more frequently found were similar after infection with attenuated and wild-type viruses, indicating that the attenuation process might not have changed the tissue tropism of the viruses substantially, with the notable exception of the lung. Thus, the percentage of positive lungs after wild-type PRRSV infection is typically close to 100% during the acute phase of the infection [5,28], while in the present study the percentage of positive lung samples ranged from 20% to 66.7%. As previously stated, the fact that replication of the vaccine strains is specially impaired in the lung compared to other organic locations seems to indicate that during PRRSV attenuation by successive passages in established cell lines, it can lose, at least partially, its ability to replicate in the cell type that constitutes its target cell in the lung, specifically, the alveolar macrophage [29,30].

The lower replication of vaccine viruses compared to wild-type viruses in different organic locations might reduce antigenic presentation, which might have consequences on the development of a potent immune response. Particularly, the low replication rate in the lung might poorly stimulate the development of specific immune response in the main target organ of PRRSV and at mucosal sites. However, specific studies to demonstrate whether attenuation and its subsequent effects in organic replication of the vaccine viruses have any influence on the development of specific immune response should be carried out because, to our knowledge, no information is available on this topic. Nonetheless, in most of the studies carried out to determine the protection elicited by different MLV PRRSV vaccines, the results show that partial protection is the typical outcome upon challenge of vaccinated pigs with different virulent isolates [17,31,32], which is not significantly different from the expected cross-protection between wild-type PRRS viruses [33]. Consequently, it seems that impaired replication of vaccine viruses in the host is not clearly related to the induction of a poor immune response and limited protection. On the contrary, PRRSV variability seems to play a major role in the lack of complete protection between PRRSV isolates [33,34].

Finally, one of the parameters used to determine the safety of MLV vaccines is the capability of the vaccine strains to be shed by vaccinates and infect non-vaccinated in-contact animals. In fact, the most significant difference between the MLV vaccine strains used in this study is their capability to be transmitted to sentinel pigs housed in direct contact with vaccinates. Thus, the vaccine strains Ingelvac PRRS and Amervac PRRS were transmitted to 100% and 88.9% of the sentinel pigs, respectively, while vaccine strains Porcilis PRRS and Pyrsvac-183 were found in only 44.4% and 22.2% of in-contact pigs, respectively. These differences were statistically significant when the results of groups A and B were compared to those of Group C and when the results of Group A were compared to those of Group D. These data seem to be inconsistent with the relatively homogenous shedding pattern by different routes found in vaccinated animals, regardless of the vaccine strain used in the immunization, and with the rather low frequency of detection of vaccine viruses in the different secretions analyzed (8.0%, 5.33%, 6.67% and 4.89% for groups A, B, C and D, respectively).

These differences do not justify, at least completely, the clear differences found in the transmission rate between the different experimental groups. Other factors, like differences in cellular tropism, may play a role in the final transmission capacity of a vaccine strain. In this line of thinking, it should be kept in mind that the route of exposure of sentinel pigs to the vaccine viruses shed by vaccinated pigs is most likely the oro-nasal route. Thus, only viruses able to infect a pig by this route will be able to establish infection. Therefore, there might be viruses capable of establishing an infection when they are injected to the pig by the IM route that have difficulties in reaching cells susceptible to infection when they reach the oro-nasal mucosa of the pig in the portal of entry into the host. The theory that not all vaccine viruses contained in a vaccine dose might be able to infect a pig by the oro-nasal route is supported by the results of a previous study carried out in our laboratory in which not all growing pigs exposed to 10^5^ TCID_50_ of different vaccine strains by IN route got infected, with the notable exception of pigs exposed to the vaccine strain Ingelvac PRRS (unpublished results).

Coherently with these findings, it is expected that those viruses with a broader range of target cells will be more efficient in the infection of susceptible pigs by different routes than those exhibiting a narrower range of target cells. This hypothesis is supported by the fact that the vaccine strains with lower capacity of replication in vivo, and apparently unable to replicate in MAP cells, were transmitted to a lower proportion of in-contact pigs (i.e. groups C and D). In contrast, vaccine strains that retained, although significantly impaired, their ability to replicate in MAP cells and were more extensively distributed in vaccinated pigs, were transmitted to a higher proportion of susceptible sentinels. Even more, when the capacity of vaccine strain shedding by the infected sentinel pigs was studied, it was found that the frequency of shedding varied depending on the group considered. Thus, the shedding rate by infected sentinel pigs of Group A was higher than by vaccinated pigs of any of the experimental groups and sentinel pigs of all other groups. In addition, the shedding rate was similar between vaccinated and infected sentinel pigs of Group B. On the contrary, the shedding rate by infected sentinels of groups C and D was lower than by vaccinated pigs of the same groups. These differences, although not statistically significant, might indicate that the vaccine strain that was the most frequently shed and transmitted to in-contact pigs retained its ability to be shed, and maybe transmitted to further pigs, after replication in non-vaccinated pigs. On the contrary, the shedding of vaccine strains by sentinels of groups C and D was poor and restricted to a low number of pigs. Thus, the data presented point towards the maintenance of some of the vaccine strains in the pig population and towards the dying out of the infection by others. However, these results should be considered carefully, especially since the replication rate (*R* value) could not be estimated in this study. This was due to limitations in the experimental design of the study that focused mainly on the determination of the residual virulence of the vaccine strains compared (measured by their ability to cause disease and lesions in vaccinated pigs and by their organic distribution) and not only on the transmission capability of each vaccine strain.

Although clinical signs were never observed in infected sentinel pigs of any group, indicating that vaccine strains had not significantly changed their virulence after one *in vivo* passage, specific studies to determine the transmissibility of each vaccine strain should be designed in the future. This is so because the transmission capability of a vaccine strain might be very relevant to its safety due to the fact that the maintenance of a vaccine virus in a population might increase the risk of reversion to virulence. In fact, it has been suggested that genetic reversion to virulence is a gradual process occurring with prolonged passage time of a vaccine strain in the field [16]. This process might be facilitated in the case of PRRSV by its high mutation rate [35,36]. Consistent with this theory is the description of virulent revertants of some vaccine strains in different countries. Although some of those viruses have a limited virulence [37] in other cases revertants are able to cause clinical signs in experimentally infected pigs [38].

In the light of these results, and despite the fact that infected sentinel pigs did not show any clinical sign, it would be sensible to prevent, as much as possible, direct contact between recently vaccinated and unvaccinated/susceptible animals under farm conditions to avoid the possibility of vaccine virus transmitting from pig-to-pig. Thus, it would be advisable to vaccinate at the same time all animals in a batch and keep them in isolation the following weeks, avoiding contact with non-vaccinated pigs. This relatively simple management measure might help to avoid the risk of transmission to in-contact pigs that seems to be inherent to PRRSV vaccine strains.

In conclusion, all vaccine strains compared in this study can be considered clinically safe. However, some differences were found in virological parameters. The onset of viremia was delayed in pigs of groups C and D, indicating some difficulties in the initial *in vivo* replication of these vaccine viruses. Besides, they were not detected in PAM cultures or in lung sections by IHC, indicating that these viruses might have lost their ability to replicate in PAM cells. This reduced ability to replicate in PAM cells found in this study might be related to the lower transmission rate recorded for these two vaccine strains, maybe due to a difficulty in establishing infection when these viruses reach the mucosal surfaces of the host.

## Competing interests

The authors declare that they have no competing interests.

## Authors’ contributions

FJM participated in recording of clinical signs and lesions, necropsy of pigs, determination of PRRSV presence by virus isolation, analysis of data (including statistical analysis) and drafting of the manuscript. LC participated in determination of PRRSV presence in clinical samples, determination of seroconversion and analysis of data. FD participated in the recording of clinical signs and the determination of PRRSV by RT-PCR in different samples. JS performed the histopathological studies and contributed to drafting the manuscript. CG and IS participated in the recording of clinical signs and necropsies. JMC participated in the design of the experiment and drafting of the manuscript. CP participated in the design of the experiment, recording of clinical signs, necropsies, analysis of data and drafting of the manuscript. All authors read and approved the final manuscript.

## References

[B1] Mengeling WL Lager KM Vorwald AC Temporal characterization of transplacental infection of porcine fetuses with porcine reproductive and respiratory syndrome virus Am J Vet Res 1994 44 1391 1398 7998696

[B2] Done SH Paton DJ White ME Porcine reproductive and respiratory syndrome virus (PRRSV): a review with emphasis on pathological, virological and diagnostic aspects Br Vet J 1996 44 153 174 10.1016/S0007-1935(96)80071-6 8680839 PMC7130409

[B3] Cavanagh D Nidovirales: a new order comprising Coronaviridae and Arteriviridae Arch Virol 1997 44 629 633 9349308

[B4] Halbur PG Paul PS Frey ML Landgraf J Eernisse K Meng XJ Lum MA Andrews JJ Rathje JA Comparison of the pathogenicity of two US porcine reproductive and respiratory syndrome virus isolates with that of the Lelystad virus Vet Pathol 1995 44 648 660 10.1177/030098589503200606 8592800

[B5] Martínez-Lobo FJ Díez-Fuertes F Segalés J García-Artiga C Simarro I Castro JM Prieto C Comparative pathogenicity of type 1 and type 2 isolates of porcine reproductive and respiratory syndrome virus (PRRSV) in a young pig infection model Vet Microbiol 2011 44 58 68 10.1016/j.vetmic.2011.06.025 21831539

[B6] Bautista EM Goyal SM Collins JE Serologic survey for Lelystad and VR-2332 strains of porcine respiratory and reproductive syndrome (PRRS) virus in US swine herds J Vet Diagn Invest 1993 44 612 614 10.1177/104063879300500418 8286463

[B7] Nelson EA Christopher-Hennings J Drew T Wensvoort G Collins JE Benfield DA Differentiation of U.S. and European isolates of porcine reproductive and respiratory syndrome virus by monoclonal antibodies J Clin Microbiol 1993 44 3184 3189 7508455 10.1128/jcm.31.12.3184-3189.1993 PMC266373

[B8] Meng XJ Paul PS Halbur PG Lum MA Phylogenetic analyses of the putative M (ORF 6) and N (ORF 7) genes of porcine reproductive and respiratory syndrome virus (PRRSV): implication for the existence of two genotypes of PRRSV in the U.S.A. and Europe Arch Virol 1995 44 745 755 10.1007/BF01309962 7794115 PMC7086766

[B9] Gorcyca D Schlesinger K Chladek D Behan W Polson D Roof M Doitchenoff D RespPRRS: a new tool for the prevention and control of PRRS in pigs 1995 Omaha, Nebraska USA: Proceedings of the 26th Ann Meet Am Assoc Swine Pract: 4–7 March 1995 1 22

[B10] Lager KM Mengeling WL Current status of vaccines and vaccination for porcine reproductive and respiratory syndrome 1997 Quebec City, Quebec, Canada: Proceedings of the 28th Ann Meet Am Assoc Swine Pract: 1–4 March 1997 443 446

[B11] Mengeling WL Lager KM Wesley RD Clouser DF Vorwald AC Roof MB Diagnostic implications of concurrent inoculation with attenuated and virulent strains of porcine reproductive and respiratory syndrome virus Am J Vet Res 1999 44 119 122 9918159

[B12] Mengeling WL Lager KM Vorwald AC Edited by Veterinary Outreach Programs: University of Minnesota An overview on vaccination for porcine reproductive and respiratory syndrome In Proceedings of AD Leman Swine Conf: September 1996 1996 Saint Paul, Minnesota, USA 139 142

[B13] Mengeling WL Vorwald AC Lager KM Brockmeier SL Comparison among strains of porcine reproductive and respiratory syndrome virus for their ability to cause reproductive failure Am J Vet Res 1996 44 834 839 8725809

[B14] Mengeling WL Lager KM Vorwald AC Clinical effects of porcine reproductive and respiratory syndrome virus on pigs during the early postnatal interval Am J Vet Res 1998 44 52 55 9442243

[B15] Bøtner A Strandbygaard B Sørensen KJ Have P Madsen KG Madsen ES Alexandersen S Appearance of acute PRRS-like symptoms in sow herds after vaccination with a modified live PRRS vaccine Vet Rec 1997 44 497 499 10.1136/vr.141.19.497 9402722

[B16] Mengeling WL Vorwald AC Lager KM Clouser DF Wesley RD Identification and clinical assessment of suspected vaccine-related field strains of porcine reproductive and respiratory syndrome virus Am J Vet Res 1999 44 334 340 10188816

[B17] Prieto C Alvarez E Martínez-Lobo FJ Simarro I Castro JM Similarity of European porcine reproductive and respiratory syndrome virus strains to vaccine strain is not necessarily predictive of the degree of protective immunity conferred Vet J 2008 44 356 363 10.1016/j.tvjl.2007.01.021 17560818

[B18] Scortti M Prieto C Martínez-Lobo FJ Simarro I Castro JM Effects of two commercial European modified-live vaccines against porcine reproductive and respiratory syndrome viruses in pregnant gilts Vet J 2006 44 506 514 10.1016/j.tvjl.2005.07.015 16169756

[B19] Shi M Lam TT Hon CC Hui RK Faaberg KS Wennblom T Murtaugh MP Stadejek T Leung FC Molecular epidemiology of PRRSV: a phylogenetic perspective Virus Res 2010 44 7 17 10.1016/j.virusres.2010.08.014 20837072

[B20] Kim HS Kwang J Yoon IJ Joo HS Frey ML Enhanced replication of porcine reproductive and respiratory syndrome (PRRS) virus in a homogeneous subpopulation of MA-104 cell line Arch Virol 1993 44 477 483 10.1007/BF01313785 8257302

[B21] Prieto C Suárez P Simarro I García C Fernández A Castro JM Transplacental infection following exposure of gilts to porcine reproductive and respiratory syndrome virus at the onset of gestation Vet Microbiol 1997 44 301 311 10.1016/S0378-1135(97)00112-0 9444067

[B22] Suárez P Zardoya R Prieto C Solana A Tabarés E Bautista JM Castro JM Direct detection of the porcine reproductive and respiratory syndrome (PRRS) virus by reverse polymerase chain reaction (RT-PCR) Arch Virol 1994 44 89 99 10.1007/BF01309767 7545931

[B23] Reed JJ Muench RH A simple method to estimating fifty percent end points Am J Hyg 1938 44 493 497

[B24] Halbur PG Paul PS Frey ML Landgraf J Eernisse K Meng XJ Andrews JJ Lum MA Rathje JA Comparison of the antigen distribution of two US porcine reproductive and respiratory syndrome virus isolates with that of the Lelystad virus Vet Pathol 1996 44 159 170 10.1177/030098589603300205 8801709

[B25] Hesse RA Couture LP Lau ML Dimmick BS Ellsworth SR Efficacy of Prime Pac PRRS in controlling PRRS reproductive disease: homologous challenge 1996 Nashville, Tennessee, USA: Proceedings of the 27th Ann Meet Am Assoc Swine Pract: 2–5 March 1996 103 105

[B26] Halbur PG Paul PS Meng XJ Lum MA Andrews JJ Rathje JA Comparative pathogenicity of nine US porcine reproductive and respiratory syndrome virus (PRRSV) isolates in a five-week-old cesarean-derived, colostrum-deprived pig model J Vet Diagn Invest 1996 44 11 20 10.1177/104063879600800103 9026065

[B27] Rossow KD Collins JE Goyal SM Nelson EA Christopher-Hennings J Benfield DA Pathogenesis of porcine reproductive and respiratory syndrome virus infection in gnotobiotic pigs Vet Pathol 1995 44 361 373 10.1177/030098589503200404 7483210

[B28] Prieto C García C Simarro I Castro JM Temporal shedding and persistence of porcine reproductive and respiratory syndrome virus in boars Vet Rec 2004 44 824 827 10.1136/vr.154.26.824 15260446

[B29] Duan X Nauwynck HJ Pensaert MB Effects of origin and state of differentiation and activation of monocytes/macrophages on their susceptibility to porcine reproductive and respiratory syndrome virus (PRRSV) Arch Virol 1997 44 2483 2497 10.1007/s007050050256 9672608 PMC7086874

[B30] Lawson SR Rossow KD Collins JE Benfield DA Rowland RR Porcine reproductive and respiratory syndrome virus infection of gnotobiotic pigs: sites of virus replication and co-localization with MAC-387 staining at 21 days post-infection Virus Res 1997 44 105 113 10.1016/S0168-1702(97)00086-5 9498609

[B31] Scortti M Prieto C Simarro I Castro JM Reproductive performance of gilts following vaccination and subsequent heterologous challenge with European strains of porcine reproductive and respiratory syndrome virus Theriogenology 2006 44 1884 1893 10.1016/j.theriogenology.2006.04.043 16806451

[B32] Zuckermann FA Alvarez Garcia E Diaz Luque I Christopher-Hennings J Doster A Brito M Osorio F Assessment of the efficacy of commercial porcine reproductive and respiratory syndrome virus (PRRSV) vaccines based on measurement of serologic response, frequency of gamma-IFN-producing cells and virological parameters of protection upon challenge Vet Microbiol 2007 44 69 85 10.1016/j.vetmic.2007.02.009 17376612

[B33] Lager KM Mengeling WL Brockmeier SL Evaluation of protective immunity in gilts inoculated with the NADC-8 isolate of porcine reproductive and respiratory syndrome virus (PRRSV) and challenged-exposed with an antigenically distinct PRRSV isolate Am J Vet Res 1999 44 1022 1027 10451216

[B34] Labarque G Van Gutch S Van Reeth K Nauwynck H Pensaert M Respiratory tract protection upon challenge of pigs vaccinated with attenuated porcine reproductive and respiratory syndrome virus vaccines Vet Microbiol 2003 44 187 197 10.1016/S0378-1135(03)00157-3 12935746

[B35] Hanada K Suzuki Y Nakane T Hirose O Gojobori T The origin and evolution of porcine reproductive and respiratory syndrome viruses Mol Biol Evol 2005 44 1024 1031 10.1093/molbev/msi089 15659555 PMC7107557

[B36] Prieto C Vázquez A Núñez JI Alvarez E Simarro I Castro JM Influence of time on the genetic heterogeneity of Spanish porcine reproductive and respiratory syndrome virus isolates Vet J 2009 44 363 370 10.1016/j.tvjl.2008.01.005 18684650

[B37] Opriessnig T Halbur PG Yoon KJ Pogranichniy RM Harmon KM Evans R Key KF Pallares FJ Thomas P Meng XJ Comparison of molecular and biological characteristics of a modified live porcine reproductive and respiratory syndrome virus (PRRSV) vaccine (Ingelvac PRRS MLV), the parent strain of the vaccine (ATCC VR2332), ATCC VR2385, and two recent field isolates of PRRSV J Virol 2002 44 11837 11844 10.1128/JVI.76.23.11837-11844.2002 12414926 PMC136866

[B38] Kranker S Nielsen J Bille-Hansen V Bøtner A Experimental inoculation of swine at various stages of gestation with a Danish isolate of porcine reproductive and respiratory syndrome virus (PRRSV) Vet Microbiol 1998 44 21 31 10.1016/S0378-1135(98)00176-X 9646462

